# Editorial: Pathogenesis and clinical treatment of diabetic nephropathy

**DOI:** 10.3389/fmed.2025.1559841

**Published:** 2025-02-03

**Authors:** Ziyun Xu, Sheng Qiang, Zhaoyong Hu, Haiyong Chen

**Affiliations:** ^1^School of Chinese Medicine, The University of Hong Kong, Pok Fu Lam, Hong Kong SAR, China; ^2^Department of Nephrology, Zhangjiagang TCM Hospital Affiliated to Nanjing University of Chinese Medicine, Zhangjiagang, Jiangsu, China; ^3^Division of Nephrology, Baylor College of Medicine, Houston, TX, United States; ^4^Department of Chinese Medicine, The University of Hong Kong-Shenzhen Hospital, Shenzhen, China

**Keywords:** diabetic nephropathy, epigenetics, pharmacological agents, diabetic kidney disease, fracture

Diabetic Nephropathy (DN) is a severe complication of diabetes mellitus, representing a significant global health burden. It is estimated that there are 823 million diabetes patients in 2022, with the number projected to exceed 1.3 billion by 2050 (1, 2). The worldwide study indicates that the prevalence of end-stage renal disease (ESRD) is 29% among individuals with diabetes (3).

Recent research has provided insights into different aspects of the pathogenesis and clinical treatment of DN. The editorial aims to synthesize insights from four pivotal studies that collectively deepen our understanding of the pathogenesis and clinical management of DN (Bilen et al., Wei et al., Kim et al., Cheng et al.). These studies address key areas in diabetic nephropathy, including recent advances in pharmacological management, the association between body weight fluctuations and fracture risk, the evaluation of clinical efficacy and safety in peritoneal dialysis and hemodialysis patients, and the role of protein methylation in DN pathogenesis. Together, they offer a multifaceted perspective on the challenges and opportunities in managing DN.

Bilen et al. provided a comprehensive summary of the pharmacological agents currently available for the treatment of DN. These agents encompass angiotensin-converting enzyme (ACE) inhibitors, angiotensin II receptor blockers, sodium-glucose cotransporter 2 (SGLT2) inhibitors, glucagon-like peptide-1 receptor (GLP-1) agonists, mineralocorticoid receptor antagonists, and endothelin receptor antagonists. The article offers an in-depth review of the clinical applications, effectiveness, practical considerations, and scientific evidence of these agents.

Currently, no pharmacological agents can halt the progression of DN. Patients with DN typically progress to ESRD within the following decades. Hemodialysis (HD), peritoneal dialysis (PD), and kidney transplantation are common therapies for these patients. Building on the challenges of managing DN progression, Wei et al. compared efficacy and safety of the use of HD and PD for Chinese patients with ESRD through a systematic review and meta-analysis. Their findings suggest that PD outperforms HD in preserving residual kidney function, minimizing hemodynamic stress, and reducing bleeding and cardiovascular events, though it carries a higher risk of malnutrition and infection.

Declining renal function in DN exacerbates metabolic and nutritional abnormalities, including mineral and bone disorders, protein-energy malnutrition. However, it remains unclear whether changes of body weight increase the risk of fracture in DN population. Kim et al. analyzed the association in DN patients using data from a Korean nationwide cohort study. The findings revealed that both weight gain and loss elevate fracture risk in DN patients. The study underscores the importance of awareness among DN patients regarding the potential fracture risk associated with significant weight fluctuations.

Beyond clinical factors, the pathogenesis of DN is increasingly understood through molecular mechanisms. Epigenetic modifications, such as DNA methylation, histone acetylation, non-coding RNA, significantly influence protein synthesis, function and degradation, contributing to the pathogenesis of DN (4). Various studies focus on epigenetic modifications at the DNA and mRNA levels. Cheng et al. conducted a comprehensive review of histone methylations (e.g., histone lysine methylation, histone arginine methylation) and non-histone methylations in the pathogenesis of DN and highlighted pharmacological development strategies for treating DN.

Together, the articles in this Research Topic showcase diverse facets of DN, from clinical management to molecular mechanisms, collectively contributing to a more comprehensive understanding of DN and offering valuable insights for future research directions and therapeutic strategies. In conclusion, this Research Topic compiles articles that offer novel insights into the pathogenesis and clinical management of DN.
